# Allelic based gene-gene interactions in rheumatoid arthritis

**DOI:** 10.1186/1753-6561-3-S7-S76

**Published:** 2009-12-15

**Authors:** Jeesun Jung, Joon Jin Song, Deukwoo Kwon

**Affiliations:** 1 Department of Medical and Molecular Genetics, Indiana University School of Medicine, 410 West 10th Street, HITS 5000, Indianapolis, Indiana 46202, USA; 2 Department of Mathematical Sciences, University of Arkansas, 301 SCEN, Fayetteville, Arkansas 72701, USA; 3 Division of Cancer Epidemiology and Genetics, National Cancer Institute, 6120 Executive Boulevard, EPS Room 7056, Rockville, Maryland, 20852, USA

## Abstract

The detection of gene-gene interaction is an important approach to understand the etiology of rheumatoid arthritis (RA). The goal of this study is to identify gene-gene interaction of SNPs at the allelic level contributing to RA using real data sets (Problem 1) of North American Rheumatoid Arthritis Consortium (NARAC) provided by Genetic Analysis Workshop 16 (GAW16). We applied our novel method that can detect the interaction by a definition of nonrandom association of alleles that occurs when the contribution to RA of a particular allele inherited in one gene depends on a particular allele inherited at other unlinked genes. Starting with 639 single-nucleotide polymorphisms (SNPs) from 26 candidate genes, we identified ten two-way interacting genes and one case of three-way interacting genes. SNP rs2476601 on *PTPN22 *interacts with rs2306772 on *SLC22A4*, which interacts with rs881372 on *TRAF1 *and rs2900180 on *C5*, respectively. SNP rs2900180 on *C5 *interacts with rs2242720 on *RUNX1*, which interacts with rs881375 on *TRAF1*. Furthermore, rs2476601 on *PTPN22 *also interacts with three SNPs (rs2905325, rs1476482, and rs2106549) in linkage disequilibrium (LD) on *IL6*. The other three SNPs (rs2961280, rs2961283, and rs2905308) in LD on *IL6 *interact with two SNPs (rs477515 and rs2516049) on *HLA-DRB1*. SNPs rs660895 and rs532098 on *HLA-DRB1 *interact with rs2834779 and four SNPs in LD on *RUNX1*. Three-way interacting genes of rs10229203 on *IL6*, rs4816502 on *RUNX1*, and rs10818500 on *C5 *were also detected.

## Background

Rheumatoid arthritis (RA) is a complex, chronic inflammatory disease whose etiology remains unknown. It has been known that RA is a result of the complicated networks of multiple genes along with the environmental factors such as smoking. It is more common in females. Through a combined linkage and association study [1], the *HLA *gene cluster on 6p21 has been shown to have the most likely predisposing loci for RA. In addition to *HLA*, numerous genetic variants influence the pathology of RA. Unfortunately, detection of gene-gene interaction has been challenging due to an issue of high dimensionality from multi-locus combinations that require a large sample size.

**Figure 1:**
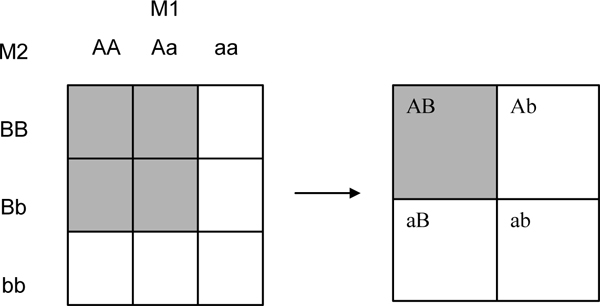
**Cell combinations of two unlinked markers; *M*_1 _has A and a alleles, and *M*_2 _has B and b alleles**.

In this study, we applied a novel approach to detect gene-gene interaction influencing RA using the case-control subjects provided by the North American Rheumatoid Arthritis Consortium (NARAC). In contrast to the previously available method searching for the interaction at the genotype level, our approach focuses on the detection of interaction of at the allelic level with a novel definition: the allele-based gene-gene interaction occurs when a particular allele in one gene and a particular allele at another unlinked genes are dependent on the contribution to RA (Figure 1) [1]. Based on the 639 SNPs from 26 candidate genes related to RA pathology, we performed a score test based on logistic regression and a *F*-test based on the Cochran-Armitage regression model developed for the detection of allelic based gene-gene interaction [2,3].

## Methods

### Characteristics of data

As a regular quality control procedure, population stratification analysis using all 531,688 single-nucleotide polymorphisms (SNPs) was performed by EIGENSTRAT as we included the subjects of the four populations from HapMap database (Yoruba, CEPH, Japanese, and Han Chinese). The result showed that the case and control subjects are confirmed as European Americans. Additionally, we tested sex inconsistency between X chromosome and the clinical report and removed seven subjects whose data were discrepant. After the removal, 866 cases and 1189 controls were used for the further analysis. The 26 candidate genes were selected based on the following reasons: 1) previous reported results (*HLA-DRB1*, *PTPN22*, and *TNF*) [4,5]; 2) genes related to macrophage migration inhibitory factor and linked to the production of inflammatory cytokines (*MIF*, *IL6*, *IL1B*, *IL3*, *IL4*, and *IL13*) [8,9]; 3) genes playing an immunologically important role in down-regulating the immune response (*CTLA4*, *RUNX1*, *STAT4*, and *SLC22A4*) [8]; 4) genes used to test for interaction by Mei et al. and Ding et al. [6,7]; 5) genes relevant to inflammatory disease [8]. We also removed SNPs deviating from Hardy-Weinberg equilibrium (*p*-value < 10^-5^) and having a minor allele frequency of less than 0.01. The names, locations, and the number of SNPs being tested for the 26 genes are provided in Table 1.

**Table 1:** List of genes selected for analysis

Gene Symbol	Locus	No. of SNPs
*TNFRSF1B*	1p36.22	20
*PADI4*	1p36.13	16
*PTPN22*	1p13.3	7
*FCGR3A*	1q23.3	1
*IL1B*	2q14	14
*ITGAV*	2q32.1	16
*STAT4*	2q32.3	35
*CTLA4*	2q33	16
*IL3*	5q31.1	3
*IL13*	5q31.1	3
*IL4*	5q31.1	4
*SLC22A4*	5q31.1	14
*HAVCR1*	5q33.3	13
*NFKBIL1*	6p21.3	7
*HLA-DRB1*	6p21.3	6
*LTA*	6p21.33	4
*TNF*	6p21.3	1
*MAP3K7IP2*	6q25.1	52
*IL6*	7p21	96
*TRAF1*	9q33	3
*C*5	9q33	27
*DLG5*	10q22.3	26
*MS4A1*	11q12.2	11
*CARD15*	16q12.1	10
*RUNX1*	21q22.12	216
*MIF*	22q11.23	18
Total		639

### Statistical model

The underlying principle of our method is to identify the association of allelic combination between two unlinked markers with a disease trait so that subjects are assigned an allelic score given their observed genotype information. The score is a conditional probability of obtaining the particular allelic combination given the observed genotypes at the two loci of each subject. For example, a subject with AA (at marker *M*_1_) and Bb (at marker *M*_2_) genotype has 1/2 in the AB combination and 1/2 in the Ab combination, *X*_*AB *_= *P*(*AB*|*M*_1 _= *AA*, *M*_2 _= *Bb*) = 1/2, *X*_*Ab *_= *P*(*Ab*|*M*_1 _= *AA*, *M*_2 _= *Bb*) = 1/2 Table 2 shows the allelic scores of a subject whose genotype is given [2].

**Table 2:** Allelic scores

	Allelic score			
	
Genotype	AB	Ab	aB	ab
(AA, BB)	1	0	0	0
(AA, Bb)	1/2	1/2	0	0
(AA, bb)	0	1	0	0
(Aa, BB)	1/2	0	1/2	0
(Aa, Bb)	1/4	1/4	1/4	1/4
(Aa, bb)	0	1/2	0	1/2
(aa, BB)	0	0	1	0
(aa, Bb)	0	0	1/2	1/2
(aa, bb)	0	0	0	1

### Score statistic by logistic regression model

Denote *y*_*i *_= 1 if *i*^th ^subject is affected by RA and *y*_*i *_= 0 otherwise. In the non-parametric maximum likelihood solution that allows an arbitrary covariate distribution, fitting a standard prospective logistic regression in case-control sampling design is equivalent to fitting a retrospective logistic regression except that an intercept in case-control sampling needs the information of sampling fraction of cases and controls [10,11]. Therefore, the prospective logistic regression model is used due to the equivalence in parameter estimates of interaction effect. The likelihood function of the standard logistic regression is

where . *X*_*i*, *AB *_= P(*AB*|*M*_1_, *M*_1_) is the allelic score of A allele and B allele from *M*_1 _and *M*_2 _genotypes of *i*^th ^subject and *β*_*k *_is interaction effect of *k*^th ^∈ {*AB*, *Ab*, *aB*} allelic combination. The overall proportion of *y *is *R*/*N*, where *R *is the number of case subjects and *N *is the number of total subjects. Under the assumption of no covariates, let *U*^*T *^= (*U*_*AB*_, *U*_*Ab*_, *U*_*aB*_)^*T *^be the score function, which is a derivative of the log-likelihood function with respect to *β *= (*β*_*AB*_, *β*_*Ab*_, *β*_*aB*_) respectively. Under the null hypothesis of no interaction effect *H*_0_: *β*_*AB *_= *β*_*Ab *_= *β*_*aB *_= 0 the efficient score test statistic under the null hypothesis is

where  and *V*^-1 ^is the submatrix of *I*^-1^(*α*, *β*_*AB*_, *β*_*Ab*_, *β*_*aB*_), which is the observed Fisher information matrix corresponding to *U*^*τ *^= (*U*_*AB*_, *U*_*Ab*_, *U*_*aB*_)^*τ*^. Detailed derivation and theoretical justification were published by Jung and Zhao [3].

### Extension of Cochran-Armitage trend regression

With the same allelic scores in Table 2 at the two markers, we can model a linear trend of proportion of cases over total number of subjects at each allelic combination, *p*_*k*, *j *_= *r*_*k*, *j*_/*n*_*k*, *j*_, where *n*_*k*, *j *_= *r*_*k*, *j*_/*s*_*k*, *j *_for *k *∈ (*AB*, *Ab*, *aB*, *ab*), *j *∈ (0,1/4,1/2,1) for two markers. *r*_*k*, *j *_and *s*_*k*, *j *_are the number of affected subjects and unaffected subjects having *j *score at *k *allelic combination, respectively. It has been shown that regressing *p*_*k*, *j *_on *Z*_*AB*, *j*_, *Z*_*Ab*, *j*_, *Z*_*aB*, *j *_is equivalent to regressing *y*_*i *_on *Z*_*AB*, *j*_, *Z*_*Ab*, *j*_, *Z*_*aB*, *j *_[12]. As an extension of Cochran-Armitage trend method, the interaction effect of two markers on RA trait can be modeled as

Under the assumption of no covariates, the theoretical regression coefficients are functions of linkage disequilibrium (LD) between a marker and a disease locus as follows:

where

,  are the average effect of the gene substitution at each disease loci and  is the magnitude of interaction effect.

The global test statistic for interaction effect over all allelic combinations under the null hypothesis *H*_0_: *β*_*AB *_= *β*_*Ab *_= *β*_*aB *_= 0 is , which follows *F*(3, *N*-4) distribution with *λ *= 0. The analytical properties of two methods were derived by Jung and Zhao [3] and simulation studies showed that the score test and *F *test by Cochran-Armitage trend are asymptotically equivalent.

### Simulation study of power and type I error rates and comparison of genotype-based method

A simulation study was performed to study power and type I error rates at the 1% significance level [3]. Six two-way interaction models were simulated using the simulation of software SNaP [3]. Most of the models were designed based on the combination of dominant and recessive inheritance at the genotypic level at each marker. These models are 1) dominant or recessive (Dom ∪ Rec), 2) recessive or recessive (Rec ∪ Rec), 3) dominant and dominant (Dom ∩ Dom), 4) dominant and recessive (Dom ∩ Rec), 5) threshold model in which the disease risk is increased when two or more high-risk alleles from either locus are present, 6) modified model in which the homozygosity at either locus confers disease risk [1]. For each model for type I error rates, we simulated 5,000 data sets. Each data set has 200 case and 200 controls under no LD between markers and disease loci. The disease risk allele frequency at each disease loci is 0.2 and the minor allele frequency of each SNP is 0.3, which is close to the real data. For power calculation, 2,000 data sets were simulated, with  at each model. The rest of parameters are the same as used for type I error rate calculation.

For comparison of genotype-based method, logistic regression of two-way interaction is modeled as follows: , where *j*, *l *= (*A*, or *a*)*m*, *n *= (*B*, or *b*) and . Additionally, we calculated the empirical power of multifactor dimensionality reduction (MDR) in the same simulated data sets. Table 3 illustrates that the power of the score test of the allelic based method over the six two-interaction models are higher than that of the two genotype-based methods. Type I error rates of the allele-based method are close to nominal value of 0.01. Further detailed results of simulation and analytical derivation are available in Jung and Zhao [3].

**Table 3:** Type I error rates and power over six two-way interaction models

	Allele-based method				Genotype-based method	
		
	Type I error rate		Power		Power	
			
Model	Score	*F*-test	Score	*F*-test	Score	MDR
Dom ∪ Rec	1.1	1.1	17.3	16.65	9.85	6.3
Modified	0.78	0.84	23.95	23.45	13.75	9.3
Dom ∩ Dom	0.8	0.84	46.75	46.15	29.3	24.5
Rec ∪ Rec	1.22	1.26	58.2	57.7	38.1	31.9
Threshold	1	1.04	92	91.75	80.45	73.45
Dom ∩ Rec	0.92	0.94	96.45	96.3	88.95	82.6

### Non-nested model comparison using Cochran-Armitage regression method

Technically, the proposed two-way interaction and three-way interaction model are not nested models, so an artificial nesting approach was employed to select the best model as follows:

Under the assumption of normal errors, an artificial nesting approach called the *J *test [14] was utilized as follows:

Because *f *is linear in *β*, the comparison requires that one estimates *δ *and then fits a linear regression and test for *α *= 0 using the ordinary *t*-statistic [13,14]. On the other hand, we can compare Akaike information criterion (AIC) and Bayesian information criterion (BIC) for a two-way model with that of a three-way interaction model.

### Analysis procedure

The procedure to search for the best interaction model consists of multiple steps based on the proposed methods.

#### Step 1

When performing a two-way interaction analysis [Model (1) and (3)] of two SNPs, each is selected from each gene and the global test for an interaction is performed. Note that two SNPs in the same gene are removed from the interaction analysis. We then compared the interaction model (3) with a main effect model (6) in order to search for the pure interacting SNPs that are not confounded with the main effects, and selected the best two-way interaction models which met three criteria: 1) the *p*-value less than 2.5 × 10^-7 ^from interaction test (the total 203,841 combination; adjusted for Bonferroni correction), 2) the *p*-value of the test for comparison between the interaction model and the main effect model less than 0.01, and 3) the testing SNPs should have both the smallest AIC and the smallest BIC. The following models were considered:

#### Step 2

Based on the pair-wise SNPs selected by Step 1, we conducted three-way interaction model analysis as we added one SNP at a time from one of the remaining genes, which is the scheme of the forward selection procedure. With the same procedure of a two-way model selection and an additional comparison of the three-way model with two-way interaction model, the best three-way interaction models were selected by the same criteria described in Step 1.

#### Step 3

We continued these steps until no further high-dimensional interaction model was identified.

## Results

**Figure 2:**
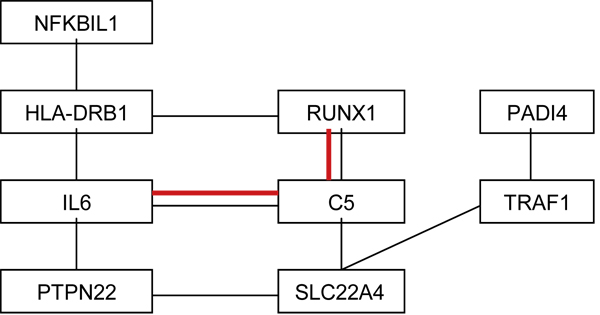
**Graphical view of the interaction of two-way and three-way interaction (red)**.

Table 4 lists ten pairs of two-way interacting genes and the function of SNPs identified. Because we analyzed all SNPs in LD with a gene, there are multiple SNPs in a gene interacting with a SNP of the other gene. SNP rs2476601 on *PTPN22 *interacts with rs2306772 on *SLC22A4*, which interacts with rs881372 on *TRAF1 *and rs2900180 on *C5*, respectively. SNP rs2900180 interacts with rs2242720 on *RUNX1*, which interacts with rs881375 on *TRAF1*. SNPs rs881375 and rs2900180 are in LD (*R*^2 ^= 0.89). Furthermore, rs2476601 on *PTPN22 *interacts with three SNPs (rs2905325, rs1476482, and rs2106549) on *IL6*. Three SNPs that are not in LD on *IL6 *interact with two SNPs (rs477515 and rs2516049) on *HLA-DRB1*. SNP rs660895 on the same gene interacts with rs2834779 on the 5' UTR region on *RUNX1*, and rs660895 and rs532098 interact with four SNPs on *RUNX1*. Additionally, rs4947324 on *NFKBIL1 *is not in LD with three SNPs on *HLA-DRB1*, but it interacts with them. Two SNPs (rs6586516 and rs2477142) on *PADI4 *interact with rs3761847 on *TRAF1*, which interacts with five SNPs in LD on *RUNX1*. Furthermore, we detected three-way interacting genes which are rs10229203 on *IL6*, rs4816502 on *RUNX1 *and rs10818500 on *C5*. Figure 2 summarized the pathway of ten pairs genes and one group of three interacting genes (indicated in red).

**Table 4:** Results of two-way (two genes) interaction and the characteristics of genes

							*p*-value^b^		
							
Gene 1 symbol (location)		Gene 2 symbol (location)	SNP1^a ^from gene 1	SNP 2^a ^from gene 2	Function of SNP 1	Function of SNP 2	Score	*F*-test	Main vs. interaction
*PADI4 *(1p36.13)	X	*TRAF1*(9q33)	rs6586516, rs2477142	rs3761847	5' UTR	5' UTR	1.66 × 10^-8^	1.43 × 10^-8^	0.005
*PTPN22 *(1p13.3)	X	*SLC22A4*(5q31.1)	rs2476601	rs2306772	Coding	intron	1.79 × 10^-12^	1.25 × 10^-12^	0.0054
*PTPN2 *(1p13.3)	X	*IL6 *(7p21)	rs2476601	rs2905325, rs1476482, rs2106549	Coding	5' UTR	3.80 × 10^-13^	2.53 × 10^-13^	0.0003
*SLC22A4 *(5q31.1)	X	*TRAF1 *(9q33)	rs2073838, rs2306772	rs881375	Intron	3' UTR	6.19 × 10^-9^	5.22 × 10^-9^	0.0037
*SLC22A4 *(5q31.1)	X	*C*5 (9q33)	rs2073838, rs2306772	rs2900180	Intron	3' UTR	1.37 × 10^-9^	1.13 × 10^-9^	0.0029
*NFKBIL1 *(6p21.3)	X	*HLA-DRB1*(6p21.3)	rs4947324^c^	rs477515, rs2516049, rs532098	3' UTR	5' UTR	<1.0 × 10^-15^	<1.0 × 10^-15^	0.0012
*HLA-DRB1 *(6p21.3)	X	*IL6 *(7p21)	rs477515, rs2516049	rs2961280, rs2961283, rs2905308	5' UTR	5' UTR	<1.0 × 10^-15^	<1.0 × 10^-15^	0.0023
*HLA-DRB1 *(6p21.3)	X	*RUNX1*(21q22.12)	rs660895	rs2834779	5' UTR	5' UTR	<1.0 × 10^-15^	<1.0 × 10^-15^	0.0028
*HLA-DRB1 *(6p21.3)	X	*RUNX1 *(21q22.12)	rs660895, rs532098	rs4817699, rs8131102, rs9984470, rs9979153	5' UTR	Intron	<1.0 × 10^-15^	<1.0 × 10^-15^	0.0037
*TRAF1 *(9q33)	X	*RUNX1 *(21q22.12)	rs881375^d^	rs4816502, rs2242720^e^	3' UTR	Intron/5' UTR	3.03 × 10^-8^	2.62 × 10^-8^	0.0027
*TRAF1 *(9q33)	X	*RUNX1 *(21q22.12)	rs3761847	rs1981392, rs2834714, rs4816502, rs2242882, rs932284	5' UTR	Intron	1.29 × 10^-11^	9.47 × 10^-12^	0.0001
*C*5 (9q33)	X	*RUNX1 *(21q22.12)	rs10760130	rs1981392, rs2834714, rs4816502, rs2242882	3' UTR	Intron	7.19 × 10^-11^	5.53 × 10^-11^	0.0001
*C*5 (9q33)	X	*RUNX1 *(21q22.12)	rs2900180^d^	rs4816502, rs2242720^e^	3' UTR	Intron/5' UTR	2.83 × 10^-10^	2.24 × 10^-10^	0.0046
*C*5 (9q33)	X	*RUNX1 *(21q22.12)	rs1468673, rs10818500	rs2834714, rs4816502	Intron	Intron	1.85 × 10^-8^	1.59 × 10^-8^	0.0001

^a^The SNPs listed under each gene are in LD (within *R*^2 ^> 0.8).

^b^The smallest *p*-value of each combination is reported.

^c^rs4947324 on *NFKBIL1 *and three SNPs (rs477515, rs2516049, rs532098) are not in LD.

^d^rs881375 on *TRAF1 *and rs2900180 on *C*5 are in LD with *r*^2 ^= 0.89.

^e^rs4816502 and rs2242720 on *RUNX1 *are not in LD, and the function of rs4817502 is intron, that of rs2242720 is 5' *UTR*, respectively.

## Discussion

In this study, the allele-based gene-gene interaction analyses were applied to case-control data sets of RA. Based on the analysis with 639 SNPs from 26 candidate genes that were previously detected through linkage study or fine mapping, we identified ten two-way interacting genes with multiple SNPs in LD from a gene and one three-way interaction. We have not identified any four-way interaction effects. However, the 26 candidate genes selected in this study may not represent all candidate genes for RA and we observed that Illumina 550k chip may not have a good gene-wide coverage for SNPs because no SNPs of *SUMO4 *and *VEGFA *in the platform are available.

A more standard interaction model using a logistic regression consisting of two main effect terms (*X *= 0, 1, 2 according to the number of alleles) and a multiplicative term of the main effect (additive × additive) was applied to the same data set. There is no interacting SNPs by an even more lenient criteria (*p*-value<10^-5^).

Three criteria to justify the significant interaction models were used. For the interaction models, Bonferroni correction was used for multiple testing, and for the comparison of the interaction model with a main-effect model (significance level of 0.01), the smaller AIC and BIC were utilized. The final selected interacting SNPs satisfied all of the criteria, which may be conservative and may cause false-negative error. There still remains the issue of multiple comparisons in the high-dimensional interactions and the complexity of the procedure to screen the interaction effects.

## Conclusion

As shown in the results, the proposed allele-based approach allows us to identify multiple interactions that may not have been identified as risk factors for RA. *PTPN22*, *SLC22A4*, *HLA-DRB1*, *IL6*, *PADI4*, *TRAF1*, *NFkBIL1*, *C5*, and *RUNX1 *may play interactive roles for RA, especially *PTPN22 *and *SLC22A4*, which are related to the reaction of antigen for RA. Therefore, our method taking into account the nonrandom association of all allelic combinations may help detect novel genetic variants and interpret biological pathways.

## List of abbreviations used

AIC: Akaike Information Criterion; BIC: Bayesian Information Criterion; GAW16: Genetic Analysis Workshop 16; LD: Linkage disequilibrium; MDR: Multifactor dimensionality reduction; NARAC: North American Rheumatoid Arthritis Consortium; RA: Rheumatoid arthritis; SNP: Single-nucleotide polymorphism

## Competing interests

The authors declare that have no competing interests.

## Authors' contributions

JJ developed statistical models, performed the analysis, and wrote the manuscript. JJS checked SAS/IML codes that JJ has written. DK contributed on the interpretation of the analysis.
